# Antenatal care practices for gestational weight gain: a cross sectional survey of antenatal care providers reported provision and barriers to providing recommended care

**DOI:** 10.1186/s12884-024-06860-x

**Published:** 2024-10-19

**Authors:** Jenna L. Hollis, Kristine Deroover, Justine Daly, Belinda Tully, Michelle Foster, Christophe Lecathelinais, Craig E. Pennell, John Wiggers, Melanie Kingsland

**Affiliations:** 1 Hunter New England Population Health, Hunter New England Local Health District, NSW Health, Locked Bag 10, Wallsend, NSW 2287 Australia; 2 Hunter Medical Research Institute, Newcastle, NSW Australia; 3 School of Medicine and Public Health, College of Health, Medicine and Wellbeing, The University of Newcastle, Callaghan, NSW Australia; 4 Hunter New England Local Health District Nursing and Midwifery Services, NSW Health, Newcastle, NSW Australia; 5 Armajun Aboriginal Health Service, Armidale, NSW Australia; 6 Gomeroi Nation New England North West, Armidale, NSW Australia

**Keywords:** Pregnancy, Diet, Physical activity, Behavioural theory, Evidenced-based practice

## Abstract

**Background:**

Implementation of recommended gestational weight gain (GWG) care by antenatal care providers is poor. It is unclear whether practice implementation and barriers differ between antenatal care provider profession or experience. This study aimed to assesses the provision of and barriers to guideline care for GWG and examine associations with professional discipline and years of experience.

**Methods:**

A cross sectional survey was conducted with antenatal care providers working in three public maternity services in a regional city in Australia. Data were collected on the provision of and barriers (informed by the Theoretical Domains Framework) to recommended GWG care. Data were summarised using descriptive statistics. Associations between health profession characteristics (professional discipline and years providing antenatal care) and GWG care practices and barrier outcomes were assessed using multivariate logistic regression.

**Results:**

117 antenatal care providers completed the survey (75% participation rate). One quarter (25%) reported that they routinely provided recommended GWG assessment at the first antenatal visit, and 9% at subsequent visits. Only 7% routinely provided recommended advice on GWG, healthy eating and physical activity. Professional discipline or years of experience were not associated with higher odds of GWG practices. Skills, belief about capabilities, belief about consequences and environmental context and resources were barriers to providing care. Medical professionals had higher odds of agreeing that they have been adequately trained to address GWG (OR = 9.14, 95%CI:3.10–26.90) and feel competent in having sensitive conversations with pregnant women about GWG (OR = 8.60, 95%CI:2.29–32.28) than midwives. Midwives had higher odds of agreeing that there are services they can refer pregnant women to for further support (OR = 2.80, 95%CI:1.13–6.91).

**Conclusions:**

The provision of antenatal care for GWG was low, inconsistently provided and did not differ by professional discipline or years of experience. Antenatal care providers report numerous barriers including skills, belief about capabilities, belief about consequences, and environmental context and resources. Barriers to GWG care provision differed by professional discipline, but not years of providing care. The findings demonstrate that the type and prioritisation of practice-change implementation strategies may need to be tailored to address the differential barriers faced by professional groups.

## Introduction

Gestational weight gain (GWG) below or above recommended levels is an international health priority given its association with poor immediate and long-term pregnancy, maternal and child health outcomes [1, 2]. Internationally, most pregnant women gain weight outside of recommended levels, with a meta-analysis of studies published between 1999 and 2017 estimating that 23% of women gain weight below, and 47% gain weight above [3] recommended GWG guidelines [4]. Similar rates of GWG above and below recommended levels have been found in Australia in a study conducted in 2015 [5], including among Australian Aboriginal women in a study conducted from 2013 [6]. Dietary intake and physical activity are two modifiable behaviours that influence GWG [1, 7].

Antenatal care services are a key setting to support pregnant women to improve dietary intake and physical activity behaviours to meet their recommended GWG range [8, 9]. In a systematic review of 117 randomised control trials, antenatal interventions that addressed diet and/or physical activity were associated with reduced GWG (− 1.15 kg; 95% CI, − 1.40 to − 0.91) and lower odds of gestational diabetes (odds ratio [OR], 0.79; 95% CI, 0.70–0.89) and total adverse maternal outcomes (OR, 0.89; 95% CI, 0.84–0.94) compared with routine care [1]. Consistent with this systematic review evidence, clinical practice guidelines from many countries, including the United States (US) [4], Canada [10], New Zealand [11] and Ireland [12] recommend that health care providers routinely weigh pregnant women and discuss recommended GWG, dietary intake and physical activity behaviours at antenatal visits [10]. In 2018, The Australian Clinical Practice Guidelines for Pregnancy Care [13] were amended to the current recommendations that at the initial and all follow-up visits, antenatal care providers weigh women; provide advice on recommended GWG, healthy eating and physical activity; and offer referrals (such as to a dietitian) for additional support if needed. Prior to this, the guidelines [14] recommended that such practices (assess, advise and refer) be provided to women during antenatal care, with repeated weighing confined to circumstances that are likely to influence clinical management.

Despite these recommendations, evidence suggests that the delivery of recommended care for GWG is less than optimal [15]. An international narrative literature review of 54 studies, including 34 quantitative and 20 qualitative studies, assessing GWG communication between health care providers and women showed that GWG care was infrequent and often inaccurate [15]. Health care providers reported provision of GWG advice ranged between 28% [16] and 95.5% [17], which was generally higher than what pregnant women reported which ranged from 9.5 to 83% [15, 18]. Although the defintion of GWG advice differed between studies, the review reported that counselling on the risks associated with discordant GWG and being advised about a target weight gain were the two most common advice components [15]. Weighing practices and frequency varied greatly between studies from 19% [19] to 92% [20] and between health care professions [15]. In a mixed methods study, including 23 interviews and a large survey of 508 healthcare providers in Canada, general practitioners (92%) reported the highest frequency of weighing, followed by obstetricians (88%), nurses (84%), and midwives (35%) [20]. These findings are consistent across research in Canada [21], US [22] and other countries around the world [15] which show inconsistent and often inaccurate advice on recommended GWG ranges, limited discussion of dietary intake and physical activity, and insufficient referral to other health professionals for additional support [15, 22, 23].

Antenatal care providers face multiple barriers to implementing recommended care for GWG [24]. A systematic review of key barriers that health professionals experience when providing maternal obesity and weight management care in pregnancy identified common barriers as a lack of knowledge of evidence-based weight recommendations and management strategies, beliefs about the consequences (e.g. that it will negatively impact their rapport with pregnant women), and a lack of supportive environmental contexts and resources, including restrictive referral pathways [24]. Care delivery and barriers to recommended GWG care are likely to differ between countries, healthcare services and models of antenatal care. There is a need to examine GWG practice implementation and contextual barriers to providing care in Australia, including the socio-cultural background of women to develop tailored approaches, including providing culturally appropriate care and referral options with Australian Aboriginal women and their families. In Australia, antenatal care providers views, knowledge and adherence to guideline recommended GWG care have been investigated in qualitative research involving relatively small sample sizes and/or in one professional discipline [23, 25, 26], with only a few studies of quantitative nature limited by low response rates [27, 28]. A comprehensive understanding of GWG practice implementation and perceived barriers by Australian antenatal care providers is needed, and it is unclear whether these factors differ between professional disciplines or experience. This study aimed to determine the provision of and barriers to guideline recommended care for GWG, examined by antenatal care profession and years of professional experience.

## Materials and methods

### Ethical approval

Ethics approval was obtained from HNELHD Human Research Ethics Committee (no. 16/10/19/5.15) and The University of Newcastle Human Research Ethics Committee (no. H-2016-0422). All methods were carried out in accordance with relevant guidelines and regulations. The study, recruitment process and survey were reviewed by Aboriginal clinicians including Aboriginal health workers and Aboriginal health practitioners working within maternity services to ensure cultural inclusion and appropriateness for Aboriginal peoples.

### Design, participants and recruitment

A cross-sectional survey was conducted with health care workers providing antenatal care in three public maternity services in a regional city in New South Wales, Australia, between November 2017 and January 2018. All health professionals (including medical staff, midwives, Aboriginal health workers, and students) who provided antenatal care at the time of the survey were eligible to participate. Two recruitment methods were used to maximise participant engagement. Firstly, potential participants were sent an invitation email via the maternity unit manager. The email included an information statement and direct link to complete the online survey. Participants had two months to consider the invitation to participate. Two reminder emails were sent to all potential participants during the study period. Secondly, potential participants were invited to participate in the survey during regular antenatal clinic meetings, daily afternoon in-services, and a monthly in-service. Participants were provided with the information statement prior to completing the survey.

#### Data collection procedures and measures

Participants were invited to complete a 25-item survey online using computers or via a paper version. Study data were collected and managed using REDCap electronic data capture tools [29, 30]. Demographic data were collected on current position, and years of experience in providing antenatal care or managing maternity services. Participants were asked to self-report their provision of guideline recommended care for GWG according to the Australian Clinical Practice Guidelines for Antenatal Care [14]. Response options were reported on a five-point Likert scale: (1) almost never (< 10% of women), (2) rarely (10–30% of women), (3) sometimes (30–60% of women), (4) often (60–90% of women) and (5) almost always (> 90% of women). Participants were also asked their knowledge of the total GWG range recommendation [4] for each pre-pregnancy BMI category (underweight, healthy weight, overweight and obesity) [31, 32]. Participants who reported that they had conducted a first antenatal visit in the previous 12 months were asked questions about their GWG practices during the first antenatal visit. Participants who reported conducting follow-up antenatal visits were asked a question relating to GWG assessment during follow-up antenatal visits.

All participants were asked survey items assessing potential barriers to guideline recommended GWG care, relating to the Theoretical Domains Framework (TDF) domains of knowledge, skills, belief about capabilities, belief about consequences, and environmental context and resources. These five domains were selected based on systematic review evidence on TDF barriers and facilitators to the implementation of pregnancy weight management and obesity guidelines [24]. The survey items were developed from an existing TDF survey [33] and a survey applying the TDF in maternity services for maternal alcohol consumption [34]. The survey was reviewed by a maternity manager to ensure clinical appropriateness prior to distribution.

### Statistical analysis

Statistical analyses were conducted using SAS, Version 9.3. Descriptive analysis reported frequencies (and percentages) of antenatal care provider characteristics, provision of guideline recommended GWG care, knowledge of GWG range recommendations, and TDF-barriers and facilitators to recommended care. Condensed antenatal care provider characteristic response categories were created for: years providing antenatal care (0–4 years; 5–9 years; 10 + years), and current position (health profession: midwives (registered midwife, clinical midwife specialist, clinical midwife educator, community liaison midwife), medical professionals (staff specialist, obstetrician, consultant, visiting medical officer, registrar, fellow, junior medical officer) and other antenatal care provider (Aboriginal Health Worker and student)).

The following GWG care delivery outcome variables were created:

- ‘*Assessment at first antenatal visit’*: reported ‘almost always (> 90% of women)’ (i) measuring a woman’s weight, (ii) asking a woman to self-report her pre-pregnancy weight, (iii) measuring a woman’s height, and (iv) calculating a woman’s BMI, at the first antenatal visit.
- *‘Assessment at follow-up antenatal visits’*: reported ‘almost always (> 90% of women)’ measuring a woman’s weight at follow-up antenatal visits.
- *‘Advise on GWG*,* healthy eating and physical activity’*: reported ‘almost always (> 90% of women)’ discussing (i) a recommended GWG range, (ii) the reason for measuring GWG, (iii) the risks of having a pre-pregnancy BMI above or below the healthy weight range, (iv) information on healthy eating and (v) information on physical activity.

Associations between health profession characteristics (health profession and years of experience) and care delivery and TDF-barrier to care provision statements were assessed using multivariable logistic regressions.

## Results

### Participants

One hundred and fifty-six antenatal care providers were invited to participate in the survey, of which 117 completed the survey (75% participation rate). Most participants reported to be midwives (66%) and to have more than 10 years of professional experience (56%). Sixty-two percent of antenatal care providers (*n* = 72) had conducted a first antenatal visit with pregnant women in the last 12 months, and 85% (*n* = 99) had conducted a follow-up antenatal visit.

### Provision of recommended care and association with health profession characteristics

One quarter of the antenatal care providers reported that they routinely provided all elements of recommended GWG assessment at the first antenatal visit (almost always; >90% of women) (Table 1). Most antenatal care providers reported calculating a pre-pregnancy BMI, however routine practices of measuring current weight and height, and collecting pre-pregnancy weight were much lower. Less than one in ten antenatal care providers reported assessing weight at the follow-up visit for almost all women.

Only 7% of antenatal care providers reported that they routinely provided all recommended elements of advice on GWG, healthy eating and physical activity at the first antenatal visit (almost always; >90% of women) (Table 1). Providing a recommended GWG range was least often provided. When asked to recall the recommended GWG ranges for women across the four BMI categories, no more than one third correctly recalled the recommended GWG ranges for women for each BMI category (Table 2).

Table 3 shows the associations between the provision of recommended care for GWG by health profession characteristics. Neither professional discipline or years of experience were not associated with higher levels of assessment at the first or follow-up antenatal visits, nor advice on GWG, healthy eating and physical activity.

**Table 1:** Provision of guideline recommended care at the first and follow-up antenatal visits

			Proportion of antenatal care providers reporting to provide care (% [95%CI])				
	Recommended GWG care practices	*N*	Almost never (< 10%)	Rarely (10–30%)	Sometimes (30–60%)	Often (60–90%)	Almost always ‘(> 90%)
First antenatal visit	Measure weight* *(not self-report)*	71	16% [8.9-25.7%]	11% [5.8-20.7%]	7% [3.1-15.5%]	11% [5.8-20.7%]	55% [43.1-66.8%]
	Ask self-reported pre-pregnancy weight	71	13% [6.8-22.4%]	4% [1.5-11.7%]	14% [7.8-24.0%]	14% [7.8-24.0%]	55% [43.1-66.8%]
	Measure height^	70	7% [3.1-15.7%]	9% [4.0-17.5%]	19% [11.2-29.2%]	10% [4.9-19.2%]	56% [43.8-67.6%]
	Calculate BMI^#^	69	3% [0.8-10.0%]	3% [0.8-10.0%]	1% [0.3-7.8%]	10% [5.0-19.5%]	83% [72.0-89.8%]
	‘*Assessment at first antenatal visit’*	72					25% [16.4-36.1%]
	Provide recommended GWG range^#^	69	20% [12.5-31.2%]	22% [13.6-32.8%]	30% [19.3-41.6%]	15% [8.1-24.7%]	13% [7.0-23.0%]
	Explain reason for measuring GWG^#^	69	10% [5.0-19.5%]	13% [7.0-23.0%]	30% [19.3-41.6%]	22% [13.6-32.8%]	25% [16.0-36.0%]
	Discuss risks of pre-pregnancy BMI^#^	69	10% [5.0-19.5%]	12% [6.0-21.3%]	39% [27.3-50.9%]	20% [12.5-31.2%]	19% [11.4-29.6%]
	Advice on healthy eating^#^	68	1% [0.3-7.8%]	6% [2.3-14.0%]	29% [18.0-40.0%]	36% [24.6-47.9%]	28% [16.7-38.4%]
	Advice on PA^#^	69	7% [3.1-15.9%]	4% [1.5-12.0%]	30% [19.3-41.6%]	38% [26.0-49.4%]	20% [12.5-31.2%]
	*‘Advise on GWG*,* healthy eating and PA’*	72					7% [3.0-15.3%]
Follow-up visit	Measure weight ‘*Assessment at follow-up antenatal visit’*	99	25% [16.5-34.0%]	28% [19.3-37.3%]	27% [18.3-36.2%]	10% [5.6-17.6%]	9% [4.9-16.4%]

Note PA = physical activity

*1 missing response; ^2 missing responses; ^#^3 missing responses

**Table 2:** Knowledge of the GWG recommendations according to pre-pregnancy BMI [4]

Pre-pregnancy BMI	*N*	Recommended GWG (kg)	Unsure	Correct	Below recommendation	Above recommendation	Irrelevant response
Underweight (≤ 18.5 kg/m^2^)*	64	12.5–18	36%	11%	39%	6%	8%
Healthy weight (18.5–24.9 kg/m^2^)*	64	11.5–16	14%	17%	56%	5%	8%
Overweight (25.0–29.9 kg/m^2^)*	64	7–11.5	44%	22%	23%	2%	9%
Obese (30.0 kg/m^2^)*	64	5–9	41%	34%	16%	3%	6%

*7 missing responses

**Table 3:** Provision of recommended GWG care by profession characteristic (health profession and number of years providing antenatal care)

Characteristics	Profession				Years providing care			
	Medical	Other provider	Midwife (referent)	*p*	0–4 years	5–9 years	10 + years (referent)	*p*
Assessment at first visit (*N* = 72)				*p* = 0.36				*p* = 0.09
n (%)	1 (11%)	2 (40%)	15 (26%)		2 (8%)	3 (38%)	13 (33%)	
Adjusted OR (95% CI)	0.50 [0.05–4.81]	4.02 [0.44–36.26]			0.16 [0.03–0.91]	1.39 [0.28–6.86]		
Assessment at follow-up visits (*N* = 99)				*p* = 0.48				*p* = 0.81
n (%)	1 (4%)	0 (0%)	8 (13%)		2 (6%)	5 (10%)	2 (13%)	
Adjusted OR (95% CI)	0.27 [0.03–2.27]	0.77 [0-4.44]			0.51 [0.06–4.15]	0.64 [0.11–3.82]		
Advice on GWG, healthy eating & physical activity (*N* = 72)				*p* = 1.00				*p* = 0.98
n (%)	0 (0%)	0 (0%)	5 (8.62%)		0 (0%)	4 (10.00%)	1 (12.50%)	
Adjusted OR (95% CI)	0.92 [0-5.56]	1.70 [0–11.00]			0.33 [0-6.33]	0.77 [0.07–8.19]		

### Antenatal care providers’ barriers and facilitators to providing recommended care and association with health profession characteristics

Most antenatal care providers agreed that pregnant women should be advised on appropriate GWG to support maternal and child health (85%; Domain = knowledge) (Table 4). However, only one quarter agreed that they had received adequate training to address GWG (24%; Domain = skills). Many identified a lack of resources and belief in their capability to provide culturally appropriate care for Aboriginal women as a barrier to providing care addressing GWG. A smaller proportion of antenatal care providers agreed that they felt competent in having conversations about GWG with pregnant Aboriginal women (45%; Domain = belief about capabilities) and were aware of culturally appropriate referrals (33%; Domain = environmental context and resources). One third of antenatal care providers believed that pregnant women would feel uncomfortable or judged if they discussed GWG with them, however only 10% believed it would have a negative effect on their professional relationship with pregnant women (Domain = belief about consequences).

**Table 4:** Antenatal care providers’ reported barriers and facilitators to guideline recommended care for GWG

TDF statement	TDF domain – definition (33, 47, 48)	*N*	Strongly agree	Agree	Neither agree nor disagree	Disagree	Strongly disagree
There is a strong rationale that pregnant women should be advised on appropriate weight gain based on pre-pregnancy body mass index (BMI) to support the health of the mother and baby.	Knowledge - An awareness of the existence of something.	111	37%	48%	11%	2%	3%
I have been adequately trained in how to address weight gain in pregnancy.	Skills (cognitive and interpersonal skills - An ability or proficiency acquired through practice. Beliefs about capabilities – perceived competence	108	5%	19%	24%	47%	5%
I feel competent in having sensitive conversations with pregnant women around weight gain in pregnancy.		108	14%	48%	24%	11%	3%
I feel competent in having sensitive conversations with pregnant Aboriginal and Torres Strait women around weight gain in pregnancy.		107	8%	37%	31%	18%	6%
Given the right resources, I am confident that I can provide advice on appropriate weight gain in pregnancy in the booking in visit.	Beliefs about capabilities - Acceptance of the truth, reality or validity about an ability, talent or facility that a person can put to constructive use.	111	24%	57%	10%	6%	3%
Regardless of how I approach the issue, pregnant women will feel uncomfortable or judged if I address weight gain in pregnancy with them during antenatal visits.	Beliefs about consequences - Acceptance of the truth, reality or validity about outcomes of a behaviour in a given situation.	111	7%	26%	25%	34%	7%
Addressing weight gain in pregnancy will have a negative effect on my relationship with pregnant women.		111	1%	9%	20%	56%	14%
I believe that the booking in visit is the most appropriate time to provide advice on appropriate weight gain in pregnancy.	Environmental context and resources - Any circumstance of a person’s situation or environment that discourages or encourages the development of skills and abilities, independence, social competence, and adaptive behaviour.	108	29%	44%	19%	7%	2%
There are support services that I can refer pregnant women to for further assistance with weight gain.		111	14%	40%	25%	16%	5%
There are culturally appropriate support services that I can refer pregnant Aboriginal and Torres Strait Islander women to for further assistance with weight gain.		109	10%	23%	37%	28%	2%

Note Theoretical Domains Framework (TDF)

Table 5 shows the results of analyses assessing associations between TDF statements on barriers to care provision and health profession characteristics. Profession was associated with two statements relating to skills and belief about capabilities, with medical professionals having higher odds of agreeing that they have been adequately trained in how to address weight gain in pregnancy (OR = 9.14, 95% CI: 3.10–26.90) and feel competent in having sensitive conversations with pregnant women around weight gain in pregnancy (OR = 8.60, 95% CI: 2.29–32.28), than midwives. For the environmental context and resources domain, midwives had higher odds than medical professionals of agreeing that there are support services that they can refer pregnant women to for further assistance with weight gain (OR = 2.80, 95% CI: 1.13–6.91). Years of providing care was not associated with any barriers to care provision.

**Table 5:** Percentages of participants that agreed or strongly agreed with the statements of the theoretical domains Framework (TDF) by health profession and number of years providing antenatal care

TDF statement		N	Profession			*p*	Years of providing care			*p*
			n (%) and Adjusted OR (95%CI)				n (%) and Adjusted OR (95%CI)			
			Medical	Other provider	Midwife		0–4 years	5–9 years	10 + years	
There is a strong rationale that pregnant women should be advised on appropriate weight gain based on pre-pregnancy body mass index (BMI) to support the health of the mother and baby	Knowledge - An awareness of the existence of something.	111	25 (86%)	8 (73%)	61 (86%)		28 (78%)	14 (93%)	52 (87%)	
			1.14 [0.32–4.08]	0.53 [0.11–2.44]		*p* = 0.65	0.57 [0.19–1.74]	2.15 [0.25–18.71]		*p* = 0.39
I have been adequately trained in how to address weight gain in pregnancy.	Skills (cognitive and interpersonal skills) - An ability or proficiency acquired through practice. Beliefs about capabilities – perceived competence	108	15 (54%)	2 (20%)	9 (13%)		7 (20%)	6 (40%)	13 (22%)	
			9.14 [3.10–26.90]	2.13 [0.36–12.57]		*p* < 0.001	0.60 [0.19–1.93]	2.49 [0.64–9.59]		*p* = 0.19
I feel competent in having sensitive conversations with pregnant women around weight gain in pregnancy.		108	25 (89%)	5 (50%)	37 (53%)		19 (54%)	11 (73%)	37 (64%)	
			8.60 [2.29–32.28]	1.14 [0.28–4.56]		*p* = 0.006	0.52 [0.20–1.36]	1.52 [0.40–5.68]		*p* = 0.25
I feel competent in having sensitive conversations with pregnant Aboriginal and Torres Strait women around weight gain in pregnancy.		107	18 (64%)	4 (40%)	27 (39%)		13 (37%)	8 (53%)	28 (49%)	
			3.14 [1.22–8.06]	1.31 [0.32–5.33]		*p* = 0.058	0.52 [0.21–1.30]	1.14 [0.35–3.68]		*p* = 0.31
Given the right resources, I am confident that I can provide advice on appropriate weight gain in pregnancy in the booking in visit.	Beliefs about capabilities - Acceptance of the truth, reality or validity about an ability, talent or facility that a person can put to constructive use.	111	25 (86%)	7 (64%)	58 (82%)		31 (86%)	13 (87%)	46 (77%)	
			1.25 [0.36–4.30]	0.31 [0.07–1.33]		*p* = 0.22	2.20 [0.67–7.19]	1.99 [0.39–10.10]		*p* = 0.36
Regardless of how I approach the issue, pregnant women will feel uncomfortable or judged if I address weight gain in pregnancy with them during antenatal visits.	Beliefs about consequences - Acceptance of the truth, reality or validity about outcomes of a behaviour in a given situation.	111	15 (52%)	5 (45%)	45 (63%)		23 (64%)	10 (67%)	32 (53%)	
			0.70 [0.28–1.72]	2.30 [0.45–11.79]		*p* = 0.37	0.86 [0.35–2.11]	1.41 [0.39–5.03]		*p* = 0.77
Addressing weight gain in pregnancy will have a negative effect on my relationship with pregnant women.		111	10 (34%)	2 (18%)	21 (30%)		11 (31%)	5 (33%)	17 (28%)	
			0.39 [0.10–1.50]	0.88 [0.09–8.66]		*p* = 0.37	0.64 [0.16–2.48]	1.35 [0.14–12.37]		*p* = 0.73
I believe that the booking in visit is the most appropriate time to provide advice on appropriate weight gain in pregnancy.	Environmental context and resources - Any circumstance of a person’s situation or environment that discourages or encourages the development of skills and abilities, independence, social competence, and adaptive behaviour.	108	22 (79%)	7 (70%)	49 (70%)		28 (80%)	11 (73%)	39 (67%)	
			1.45 [0.51–4.15]	0.81 [0.18–3.61]		*p* = 0.72	1.95 [0.70–5.43]	1.33 [0.37–4.74]		*p* = 0.44
There are support services that I can refer pregnant women to for further assistance with weight gain.		111	11 (38%)	4 (36%)	45 (63%)		18 (50%)	7 (47%)	35 (58%)	
			0.36 [0.14–0.88]	0.33 [0.09–1.28]		*p* = 0.043	0.89 [0.37–2.14]	0.64 [0.20–2.07]		*p* = 0.76
There are culturally appropriate support services that I can refer pregnant Aboriginal and Torres Strait Islander women to for further assistance with weight gain.		109	7 (24%)	4 (36%)	25 (36%)		12 (33%)	3 (20%)	21 (36%)	
			0.56 [0.21–1.52]	0.99 [0.25–3.81]		*p* = 0.51	0.94 [0.38–2.31]	0.45 [0.11–1.78]		*p* = 0.52

## Discussion

This study explored antenatal care provider practices and barriers to providing GWG care and examined differences by professional disciple and years of experience. The findings show that the majority of antenatal care providers, regardless of their professional discipline and experience, did not provide care consistent with the past [14] or current [35] Australian Clinical Practice Guidelines for Pregnancy Care. There were particularly low levels of routine care for all women (< 10% of antenatal care providers) for the recommended practices of weighing at follow-up visits and providing advice on GWG, healthy eating and physical activity. Skills, belief about capabilities, beliefs about consequences and environmental context and resources were identified as key barriers to providing GWG care, including in providing culturally appropriate care for Aboriginal women. Medical professionals had higher odds of agreeing that they had been adequately trained (domain: skills) and felt competent in conversing (domain: belief about capabilities) with pregnant women about GWG than midwives. Midwives had higher odds of agreeing that there are support services that they can refer pregnant women to for further GWG care (domain: environmental context and resources).

Most antenatal care providers in the study reported that they did not routinely (i.e., ‘almost always; > 90%) provide all elements of GWG assessment at the first or follow-up antenatal visits. The findings on infrequent weighing at follow-up antenatal visits may be influenced by the preceding Australian Clinical Practice Guidelines for Antenatal Care (2012) that recommended that repeat weighing ‘be confined to circumstances that are likely to influence clinical management’ [14]. In February 2018, the month after this study, the Australian Clinical Practice Guidelines changed to recommend routine weighing of all women at all appointments [35]. The revised Clinical Practice Guidelines were distributed via a web-based approach [35]. Systematic review evidence has shown that such passive dissemination methods are usually ineffective in supporting adherence to clinical guideline practices [36]. This is likely to have contributed to low and slow uptake of the new recommended practice of routine weighing at all visits. Governments, policy makers, clinical practice guideline developers, and health services need to employ evidenced based implementation strategies to support health care providers to uptake new care practices with changing clinical guidelines if intended population health gains are to be achieved.

Few antenatal care providers reported routinely providing all recommended advice elements of care (7%) to women, including discussing a GWG recommended range, healthy eating or physical activity at the first antenatal visit, which were recommended in both the 2012 [14] and 2018 Clinical Practice Guidelines [35]. The findings are consistent with low levels of advice found in previous research, with 22% of Australian obstetricians and midwives advising women of specific weight targets [19], compared with 15% of UK midwives [37] and 21% of Canadian prenatal healthcare providers [20]. Our study found no association between health profession characteristics and the provision of advice. This suggests that all antenatal care providers, regardless of their professional discipline or experience, encounter barriers to discussing GWG and eating and physical activity behaviours with pregnant women and require support to change their practices.

The findings show that antenatal care providers know there is a strong rationale to provide GWG care, however, they face individual and organisational barriers in doing so. Key barriers include inadequate skills and belief in their capabilities to address GWG, particularly with pregnant Aboriginal women; a belief that the consequence of providing care will make some pregnant women feel uncomfortable or judged; and environmental context and resourcing challenges with a lack of known culturally appropriate referral pathways. Such barriers are consistent with existing evidence [15, 21, 23, 24], including previous research using the Theoretical Domains Framework (TDF) [24], which showed a reluctance by antenatal care providers to discuss GWG due to a perceived lack of knowledge and skills, a belief that care may have negative consequences on their patient relationships, and an unsupportive environment and lack of resources [24]. Midwives were more likely to report inadequate training and lower competence in their communication skills as barriers than medical professionals. While a need for training to address weight management is consistent with past research [19, 24, 38–41], the difference in perceived competence in communication skills between midwives and medical professionals is a novel finding. The general training and role of communication skills in medical curriculum may result in a higher perceived ability among medical professionals to have such conversations [42]. Medical professionals reported a lack of appropriate referral options as a greater barrier than midwives, which may relate to a lack of knowledge of local community referral pathways. The findings indicate that the type and prioritisation of practice-change implementation strategies need to be tailored to address the common and differential barriers faced by professional groups [43], including to provide culturally appropriate care and referral options with Australian Aboriginal pregnant women and their families.

This evidence informs the selection of implementation strategies to improve GWG care. The Behaviour Change Wheel [44] can be used to map the barriers to the COM-B, intervention functions and behaviour change techniques. Training, education, persuasion, modelling, environmental restructuring, and enablement are evidenced-based implementation strategies [44] that address key barriers reported by antenatal care providers in this study. Systematic review evidence has shown that use of multiple implementation strategies result in increases in guideline adherence of up to 60% [36]. Individually, the use of interactive education strategies can result in improvements of 1–39% [36]. Face-to-face and/or online interactive training and education for antenatal care providers with instruction, demonstration and practice of recommended GWG care and behaviour change communication skills may be delivered by a local champion (i.e., clinical midwife educator) to address skill deficits, model recommended practices and provide social support [44]. Local data and testimonials on women’s acceptability of receiving GWG care could provide information on the positive social and emotional consequences of providing care [44]. Embedding training within antenatal services’ existing professional learning systems could aid sustainability as high staff turnover is a major barrier in maintaining practice implementation in clinical settings [45]. Clinical reminder and decision support systems can result in improvements in guideline adherence of 8-71.8% [36]. Restructuring the physical environment by modifying electronic medical record systems may enable recommended GWG care through clear instruction on how to assess, advise and refer within the context of each maternity service and the local culturally appropriate referral services available [44]. It may also remind antenatal care providers to deliver each care element and track GWG at the point-of-care [44].

Several limitations need to be considered when interpreting the findings of this study. While the study achieved a high participation rate, antenatal care providers were recruited from three maternity service so the findings may not be generalizable to other maternity services in Australia and internationally. Further, few Aboriginal health workers or student health professionals completed the survey, therefore the findings should be interpreted with such consideration. These limitations could be addressed in future research by conducting surveys across a range of maternity settings, professions, and regions to ensure appropriate representation of all services and health care providers of antenatal care. The use of self-reported data is inherently subject to limitations such as recall bias and social desirability bias [46]. While the survey items to assess barriers and facilitators to recommended GWG care were based on a validated TDF instrument [33], for the practical reason of having limited time (5–10 min) in meetings for antenatal service providers to complete the survey, we adapted the full survey to use only a selection of items and these were not re-validated. Further qualitative research would provide more in-depth and contextual information to complement these quantitative survey measures to support co-design of the implementation strategies.

## Conclusions

Antenatal care providers acknowledged that providing GWG care is important to support maternal and child health. However, provision of such care was suboptimal and antenatal care providers report numerous barriers to implementing guideline recommended GWG practices. The intended benefits of the clinical practice guidelines are unlikely to be achieved if the guidelines are not routinely implemented in antenatal care. There is a need for a multi-strategic service-wide initiative to increase assessment and care for pregnant women to address GWG, healthy eating and physical activity. The information gathered in this study is the first step in the process of identifying, refining, and testing evidenced-based implementation strategies to support antenatal care providers to provide recommended, culturally appropriate care for GWG.

## Data Availability

Data are available from the authors upon reasonable request and with permission from the Hunter New England Human Research Ethics Committee, and the University of Newcastle Human Research Ethics Committee.

## Abbreviations

BMI: Body Mass Index
GWG: Gestational weight gain
OR: Odds Ratio
